# Psychometric properties of the mammography self-efficacy and fear of breast cancer scales in Iranian women

**DOI:** 10.1186/s12889-017-4404-7

**Published:** 2017-05-31

**Authors:** Mahdi Moshki, Shole Shahgheibi, Parvaneh Taymoori, Amjad Moradi, Deam Roshani, Cheryl L. Holt

**Affiliations:** 1 Department of Public Health, School of Health Sciences; Social Development & Health Promotion Research Center, Gonabad University of Medical Sciences, Gonabad, Iran; 2 Department of Obstetrics and Gynecology, Faculty of Medicine, Kurdistan University of Medical Sciences, Sanandaj, Iran; 3 Social Determinants of Health Research Center, Kurdistan University of Medical Sciences, Sanandaj, Iran; 4 Student Research Committee, Kurdistan University of Medical Sciences, Sanandaj, Iran; 5 Department of Behavioral and Community Health, Center for Health Behavior Research, University of Maryland School of Public Health, Maryland, USA

**Keywords:** Psychometric properties, Mammography, Self-efficacy, Breast cancer fear, Iranian women

## Abstract

**Background:**

Research investigating mammography screening has often reported low mammography self-efficacy and breast cancer fear contribute to underutilization of mammography. This study aimed to examine the reliability and validity of Champion’s Mammography Self-efficacy Scale (CMSS) and Champion’s Breast Cancer Fear Scale (CBCFS) in a sample of Iranian women.

**Methods:**

The adapted instruments were administered to a community sample of 482 women aged 40 years or older. They inhibited in Sanandaj, Iran. Cronbach’s α coefficients, item-total, and test-retest correlations were used to assess the reliability of the scales. Confirmatory factor analysis was applied to assess construct validity.

**Results:**

The α coefficients for the Farsi 14-item CMSS and 8-item BCFS were .87 and.95, respectively. In terms of the CMSS confirmatory factor analysis, the proportion of *x* ^2^/df was 2.4, Comparative Fit Index (CFI) = 0.93, Tucker Lewis Index (TLI) = 0.96 providing a strong fit to the data induced by two-factor model. With regard to CBCFS, *x* ^2^/df was 86.33, CFI =0.99, and TLI =0.99 supporting one-factor model.

**Conclusion:**

The CMSS and CBCFS exhibited strong initial psychometric properties; therefore, they are recommended to understand women’s breast cancer screening behaviors better.

## Background

It is believed that the higher mortality and lower 5-years survival rate for breast cancer in Iranian women [1] might arise from inadequate use of mammography as a screening method resulting in detection of larger tumors at later stages. There is no nationwide population-based breast cancer screening program in Iran. Women who participate in routine screenings have been found to be diagnosed with earlier stage disease by 5–13 percentage points, regardless of race/ethnicity [2]. Improvements in screening practices have been shown to reduce breast cancer mortality by approximately 20–35% in women aged 50–69 and by approximately 20% in women aged 40–49 [3]. In spite of medical recommendations, many women do not receive regular mammograms in Iran [4, 5]. Increasing mammography use might be the most effective population wide approach to reduce the morbidity and mortality associated with breast cancer [6].

Research has found that variables consistently associated with breast cancer screening behaviors among Iranian women include perceived susceptibility [7], anticipated positive outcomes [8], perceived self-efficacy [4], and barriers [5]. Perceived self-efficacy for mammography has also been predictive of mammography screening in several studies [5, 9]. The aforementioned research has emphasized the role of self-efficacy in the practice of women regarding mammography. Perceived self-efficacy is defined as people’s beliefs in their capabilities to achieve given outcomes [10]. In terms of mammography, self-efficacy leads to the increased confidence among women to engage in the steps necessary for obtaining a mammogram (e.g., finding a mammography service provider, obtaining a referral, paying for the test, and preparation for getting a mammogram).

Self-efficacy may be considered a universal construct, which means that it characterizes a basic belief that is inherent in all individuals. Therefore, a cross-cultural commonality in beliefs about efficacy to produce effects by personal action might be expected [11]. Accordingly, it might be assumed that associations between self-efficacy and related constructs could be similar across cultural groups. However, without validated measures of self-efficacy (in the present context, breast cancer screening self-efficacy), assumptions around any cross-cultural differences or similarities cannot be adequately tested.

There are associations between self-efficacy and other related constructs that might influence mammography behaviors. For example, it has been shown that women with a moderate level of breast cancer fear, in combination with a higher level of self-efficacy for mammography will have greater mammography adherence than women who experience a high level of breast cancer fear in combination with lower self-efficacy for mammography [12]. Controversial findings have been reported in studies focusing on fear as a factor influencing breast screening behavior. Fear about breast cancer has been shown to reduce participation in breast cancer screening [13]. This includes worry, fear of having cancer, and fear of the screening procedure [14]. According to Champion et al., different operational definitions have been used for fear of breast cancer, resulting in these conflicting results [13]. Theoretically, whether or not an individual engages in a health behavior depends upon his or her level of fear, self-efficacy, and beliefs in the benefits of the behavior. Lower levels of self-efficacy are theorized to be associated with greater breast cancer fear [12, 13, 15]. Women who experience a very high level of breast cancer fear, especially in combination with lower self-efficacy [12, 13, 16] and fewer perceived benefits of mammography are reported to use mammography less often [17]. Women experiencing a low level of breast cancer fear, regardless of self-efficacy and perceived benefits of mammography may not feel motivated to take action. The Breast Cancer Fear Scale was developed by Champion and colleagues to assess the psychological stimulus and subjective dimensions of the breast cancer fear construct. The scale comprises items that evaluate emotional and physiological factors affecting the threat of breast cancer [13].

To date, instruments that have been utilized to evaluate a relationship between self-efficacy and mammography contained statements related to the mammography procedure [13, 18–20]. However, it is necessary for a comprehensive instrument assessing self-efficacy for mammography to cover all the steps involved in the behavior. One of the instruments that embodies this characteristic has been developed by Champion and colleagues [18] .

The concepts of breast cancer fear and self-efficacy are potentially important predictors of compliance with mammography. However, assessments of these constructs are not available for use among Iranian women across different cultures. While the psychometric properties of the mammography self-efficacy scale were previously evaluated by Hashemian in Sabzevar [20], the current work was conducted in Sanandaj, a city in the west of Iran, which is culturally and religiously different from Sabzevar. It is expected that cultural and religious factors present in Sanandaj would produce differences in the mammography self-efficacy items than when the scale was administered in Sabzevar [20].

This paper aimed to describe the cultural adaptation and validation of Farsi versions of Champion’s Mammography Self-efficacy Scale (CMSS) and Breast Cancer Fear Scale (CBCFS) [13]. These measurement tools offer the potential for more comprehensive understanding of the relationship between fear and self-efficacy with mammography adherence by providing clear conceptual and operational definitions and validated instruments.

## Methods

### Study design

We used strategies for translation and cultural adaptation of scales recommended by the World Health Organization [21]. This method involves several steps. In the first stage, the English version of the (CMSS) and (CBCFS) were given to two professional translators to translate into Farsi independently. Then, two bilinguals converted the translated instrument into the original language (English) to assure retention of the original meaning. Then, we made a comparison between the original and back-translated versions to identify any differences. In cases where differences were observed, the investigators and translators worked together to make necessary changes in the Farsi translation.

### Determination of content validity

Once the items were translated, we convened a panel consisting of two experts in health education, three gynecologists, a midwife, a radiologist, and a psychologist. The panel members were asked to review the translated items for each scale and evaluate the items’ relevance to Iranian culture, appropriateness for Iranian use, and clarity, together constituting the items’ CVI (Content Validity Index). Experts were also asked to evaluate each item on a four-point scale: 4 = very relevant, 3 = relevant with some adjustment to phrasing, 2 = only relevant if phrasing is profoundly adjusted and 1 = irrelevant. For each item, experts could propose improvements in wording. The CVI was computed as the number of experts giving a rating of either 3 or 4, divided by the number of experts—that is, the proportion in agreement about relevance. The CVI scale’s score of greater than 0.79 was considered to confirm the content validity of the scale [21].

### Mammography Self-efficacy Scale (MSS)

The instrument we adapted was based on the CMSS [18]. This is a self-report instrument that includes 10 items which loaded on 1 factor, all scored on a Likert-type scale ranging from 1 (strongly disagree) to 5 (strongly agree). The expert panel believed that 9 of the items should remain in the instrument. One item was recommended for exclusion: "If I really want to get a mammogram, I can do it” because it’s content in Farsi was repeated in item 3: “I can arrange other things in my life to have a mammogram”.

The lack of perceived capability in women to request a mammogram referral from their physicians was reported as a barrier in previous studies in Iran [22–24]. There is no routine recommendation for getting mammogram [25] and in most cases] women do not get mammography unless they request it. In addition, there are no regular healthcare visits recommended for Iranian women that assess breast health. For these reasons, the expert panel suggested assessing women’s perceived efficacy to request mammography from her physician if the doctors forget to order it or neglect it. The item “I can request from my physician to prescribe getting mammogram” was added.

According to experts’ recommendation to measure the extent to which one’s self-efficacy is unaffected by others’ support, the following question was added: “Even if nobody comes to me, I can go by myself to mammography center.” There was an agreement between experts that barriers to mammography for Iranian women include mammography being painful, feelings of shame associated with taking clothes off during mammography, old age, and having a family history of breast cancer. Therefore, the items “Even if I know that mammography is a little painful, I can do it”,” I can take clothes off and not be ashamed of it”, “I can still get mammograms despite being older”, and “Even if I have a family history of breast cancer, I can get a mammogram” were added to the scale. The CVI for the MSS was 94.8 which indicated that the scale was suitable for use in this population.

### Breast Cancer Fear Scale (BCFS)

This instrument was based on the CBCFS which is a self-report scale that includes 8 items [13]. The items included in this scale were also reviewed by the expert panel. Because there are several overlapping meanings for the term “upset” in the in Farsi language, a psychologist on the expert panel was asked to choose the best equivalent term for the word “upset” in one of the items. The psychologist made the suggestion to use “unhappy” as the most suitable Farsi equivalent or replacement for the term “upset”. The CVI for the BCFS was 98.5 suggesting an appropriate scale for use in this group of people.

### Pilot study of the instruments

To determine if the women found the questions clear and easy to understand, a pilot study was conducted using a convenience sample of 37 Iranian women who referred to healthcare centers. The inclusion criteria for pilot study included women who aged 40 or older, did not have breast cancer, and did not breastfeed or being pregnant, having had at least 1 mammogram in the past two years, and having the ability to read and write Farsi. There were no comments regarding the questionnaire and participants revealing that the questions were very clear. For the main sample size, the aforementioned inclusion criteria were also considered.

### Sample size, and sampling method

Participants were selected from women referring to healthcare centers in Sanandaj, Iran. Since there should be at least 10 participants per each item to conduct an exploratory factor analysis [26], 241 women were targeted for the 8-item breast cancer fear scale and 15-item Farsi mammography self-efficacy scale. In accordance with a cluster random sampling method, 6 centers were randomly selected from 23 centers. From each of the centers, 44 women were randomly selected for the study. The same procedure was replicated to recruit participants for conducting a confirmatory factor analysis (CFA). Finally, a consent form about the aim of the study was given to 525 women, and 507 agreed to participate (95%). The data from 18 of these participants were not used due to missing data. The Ethical Committee of Kurdistan University of Medical Sciences approved the study. The ethics code of this study is MUK.REC.1392.63.

### Statistical Analysis

Data were analyzed using SPSS, version 20 and STATA 13 software. Independent *t tests* were performed to test relationships between demographic variables, self-efficacy, and fear*,* and x^2^ tests were used to examine relationships between demographic variables and mammography adherence. The reliability coefficient for each scale was calculated using: (a) Cronbach’s alpha, (b) corrected item-total correlations with a criteria of at least 0.30, and (c) a value below 0.10 for the change in Cronbach’s alpha when an item was dropped from the scale [26]. The stability of the scales was tested using test-retest reliability over a 2-week interval. The Kaiser-Meyer-Olkin (KMO) measure of sampling adequacy was also applied. Principal component analysis with Varimax rotation was used to extract factors using a loading criteria of 0.4 or above. The technique of estimation was Robust Maximum Likelihood. Fit indices in CFA was used to examine the fit of the model, including ratio of (*X* ^2^/df < 3), the Comparative Fit Index (CFI > 0.90), Tucker Lewis Index (TLI > 0.95), Root Mean Square Error of Approximation (RMSEA), and Standardized Root Mean Square Residual (SRMSR) where the values ranged from zero to one.

## Results

### Demographic Characteristics

The Participants’ age ranged from 40 to 70 years and the mean age was 47.35 (SD = 9.81 years). Most of the women were married (88%) and homemakers (61%) and half of them had a high school diploma or university degree (51%). With regard to health insurance, 89.6% were insured. About a third of the sample (*N* = 155) reported having had at least one mammogram in the previous 2 years. Family history of breast cancer in a first–degree relative was reported by 3% of the participants and 12% reported having a history of breast problems such as pain, nipple discharge, and/or a lump (Table 1).

**Table 1:** Characteristics of study participants (*N* =482)

*Characteristics*	*N (%)*
Age	
40-50	350 (72.6)
51-60	110 (22.8)
61 and older	22 (4.6)
Marital status	
Single	16 (3.3)
Married	424 (88.0)
Widowed	42 (8.7)
Education status	
Primary	182 (37.8)
Secondary	54 (11.2)
Diploma	120 (24.9)
Academic	126 (26.1)
Number of Children	
No child	26 (5.4)
1-3	346 (71.8)
More than 3 children	110 (22.8)
Employment status	
Homemaker	192 (60.6)
Employed	190 (39.4)
Menopause	
Yes	134 (27.8)
No	358 (72.2)
History of personal breast problem	
Yes	58 (12.0)
No	424 (88.0)
Family history of breast cancer	
Yes	14 (2.9)
No	468 (97.1)
Health insurance	
Yes	432 ( 89.6)
No	50 (10.4)
History of having mammography in past 2 y	
Yes	155 (32.4)
No	327 (67.6)

Mammography adherence was classified as a binary variable, with 0 representing having one mammogram in previous 2 years and 2 representing having two or more mammograms in previous 2 years. The *x* ^2^ test results showed health insurance (*x* ^2^ = 9.28, *df =* 3, *p* < .02), menopause status (*x* ^2^ = 8.41, *df* = 1, *p* < .006), family history of breast cancer (*x* ^2^ = 5.02, *df* = 1, *p* < .02), and a history of personal breast problem (*x* ^2^ = 33.19, *df* = 1, *p* < .001) were associated with mammography behavior. There was a significant difference in self-efficacy between women who had and had not had two or more mammograms (*t* = 5.19, *p* < .001) with women who had had a mammogram reporting higher self-efficacy scores. There was no significant difference on the fear scale in accord with mammography utilization (*t* = 1.75, *p* = .080).

### Reliability

#### Internal consistency and item-total correlation analysis

Reliability of the scales was acceptable, as assessed using the Cronbach’s alpha coefficient. The total scale coefficients were 0.87 for MSS and 0.95 for BCFS (Tables 2 and 4).

**Table 2:** Factor loadings, item analysis, and the item total correlations for the 14 items in the Farsi version of the mammography self-efficacy scale (*N*=230)

Mammography Self-Efficacy	Factor loading Factor 1	Factor loading Factor 2	Item Mean (SD)	Corrected Item/Total Correlation	α if Item Deleted
1. I can ask doctor to prescribe a mammography	0.262	**0.455**	4.00 (.665)	0.41	.87
2. I can arrange transportation to get a mammogram	0.070	**0.734**	3.78 (.831)	0.39	.87
3. I can arrange other things in my life to have a mammogram	0.445	**0.584**	3.94 (.656)	0.58	.87
4. I can get a mammogram even if I am worried	**0.598**	0.457	4.01 (.629)	0.53	.94
5. I can get a mammogram even if I do not know what to expect	0.493	**0.523**	4.02 (.651)	0.63	.86
6. I can find a way to pay for a mammogram	0.081	**0.751**	3.91 (.804)	0.51	.87
7. I can make an appointment for a mammogram	0.434	**0.708**	4.04 (.621)	0.74	.85
8. I know how to go about getting a mammogram	0.404	**0.537**	3.84 (.801)	0.56	.86
9. I can find a place to have a mammogram	0.454	**0.568**	4.05 (.691)	0.64	.86
10. Even if nobody comes to me, I myself can go to mammography center	0.314	**0.598**	3.88 (.881)	0.56	.86
11. Although I know that mammography is a little painful, I can do it	**0.830**	0.209	4.01 (.642)	0.65	.86
12. I can take clothes off and not ashamed of it when mammogram	**0.746**	0.082	3.89 (.778)	0.49	.87
13. I can still get mammograms despite of high aging	**0.796**	0.233	3.99 (.716)	0.63	.86
14. Even if I have a family history of breast cancer, I can get mammography	**0.798**	0.250	4.10 (.614)	0.66	.86
Eigenvalue	5.33	1.37			
Variance (%)	44.48	11.48			
Total variance (%)	55.96				
Cronbach α	.87				
Scale mean (SD)	47.60 (5.69)				

*Bolded indicates highest factor loadings

#### Stability

The temporal stability of the scales was assessed using test-retest reliability over a 2-week interval with a random sub-sample of 43 women. The correlation coefficients for MSS and BCFS were 0.96 and 0.85, respectively (*p* < 0.001).

### Construct Validity

#### Exploratory factor analysis, Farsi version of mammography self-efficacy scale

Before extracting factors, KMO measure of sampling adequacy and Bartlett test of sphericity were conducted to ensure that the data were suitable for factor analysis. The KMO test gave the value of 0.87 and the BTS test the value of 1247.87 (df = 66, *P* < 0.001), suggesting the suitability of the data for analysis. The principal component exploratory factor analysis showed that the 14-item self-efficacy for mammography scale showed a 2-factor solution with eigenvalues greater than 1, which together accounted for 56% of the variance. Item-total correlation values ranged from 0.39 to 0.74, which justified combining the 2 factors into 1 self-efficacy scale [27]. The item, “I can talk to people at the mammogram center about my concerns,” did not meet the factor loading criterion and hence, it was eliminated. Table 2 summarizes the range of factor loadings for the items retained for each factor as well as the eigenvalues and variance explained for each factor.

#### Confirmatory factor analysis, Farsi version of the mammography self-efficacy scale

**Fig. 1:**
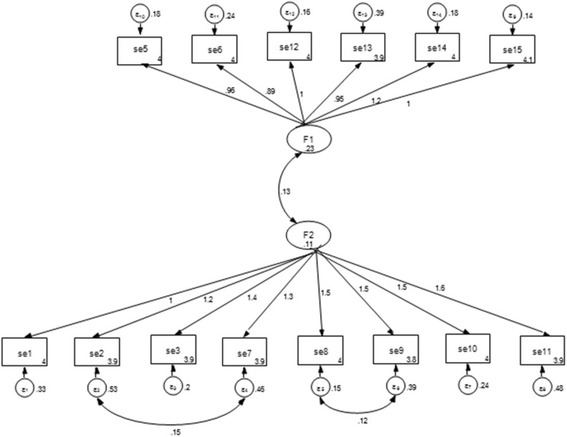
Standardized solution for the revised model based on confirmatory factor analysis for mammography self-efficacy. Numbers in circles in rectangles indicate measurement errors. *Note:* the SE 4 not entered into confirmatory factor analysis because of not meeting the factor-loading criterion

The results of confirmatory factor analysis with 14 items revealed a poor fitting model according to fit indices: *X* ^2^ = 477, CFI =0.76, TLI =0.71, RMSEA = 0.14 and SRMSR =0.24. An examination of the modification indices suggested the addition of correlated errors on item 3 pairs of items on factor 1 and 4 pairs of items on factor 2. The investigators decided to allow these correlations between errors based on the conceptual meaning of the items (SE 2), (SE 7), and (SE 3) reflecting logistics (e.g., transport, making appointment, and arranging procedures). The items (SE 8) and (SE 9) reflect the dimension of logistical steps related to the process of getting a mammogram; knowing how to obtain a mammogram, and finding the mammogram facility. In the modified model, error correlations were also set between items (SE 12) and (SE 13) related to performance magnitude (shame and aging). The modification index suggested a correlated error term between items (SE 5) and (SE 14) which both were related to being unaware of what to expect. In the modified model, the *×* ^2^ value decreased compared to the value in the initial model (*x* ^2^ /df = 2.4 that was less than 3 indicating good fit, *× 2* = 168, CFI = 0.93, TLI = 0.96, RMSEA =0.05 and SRMSR =0.07 (Table 3; Fig. 1).

**Table 3:** The fit indexes of the initial and revised model of the confirmatory factor analyses for Farsi version of the mammography self-efficacy scale (*N*=252)

	Indexes Values						
	*x* ^2^	df	*x* ^2^/df	CFI	TLI	RMSEA	SRMSR
Initial mode	447	77	5.8	0.76	0.71	0.14	0.24
Revised model	168	69	2.4	0.93	0.96	0.07	0.05

*Abbreviations: CFI* Comparative Fit Index, *RMSEA* Root Mean Square Error of Approximation, *SRMSR* Standardized Root Mean Square Residual, *TLI* Tucker Lewis Index

#### Exploratory factor analysis, Farsi version of breast cancer fear scale

The KMO test result showed the value of 0.88 and the BTS test the value of 2039.45 (df = 28, *p* < 0.001) suggesting the suitability of the data for analysis. Exploratory factor analysis with principal components showed a single factor solution for the 8-item breast cancer fear scale, which explained 74% of the variance with factor loading values in the range of 0.64 to 0.80 representing a strong relationship between each item and the corresponding factor (Table 4).

**Table 4:** Factor loadings, item analysis, and the item total correlations for the 8 items in the Farsi version of the breast cancer fear scale (*N*=230)

**Fear**	Factor loading	Item Mean (SD)	Corrected Item/Total Correlation	α if Item Deleted
1. When I think about breast cancer, I get scared.	**0.703**	3.56 (1.15)	0.64	.95
2. When I think about breast cancer, I feel nervous.	**0.776**	3.45 (1.12)	0.73	.93
3. When I think about breast cancer, I get unhappy.	**0.759**	3.56 (1.07)	0.71	.94
4. When I think about breast cancer, I get depressed.	**0.669**	3.10 (1.19)	0.53	.94
5. When I think about breast cancer, I get Edgy.	**0.641**	2.97 (1.16)	0.69	.93
6. When I think about breast cancer, my heart beats faster.	**0.764**	3.03 (1.18)	0.71	.96
7. When I think about breast cancer, I feel uneasy.	**0.784**	3.32 (1.17)	0.74	.94
8. When I think about breast cancer, I feel anxious.	**0.805**	3.29 (1.17)	0.75	.94
Eigenvalue	5.93			
Variance (%)	74.15			
Cronbach α	.95			
Scale mean (SD)	26.29 (7.95)			

*Bolded indicates highest factor loadings

#### Confirmatory factor analysis, Farsi version of the breast cancer fear scale

**Fig. 2:**
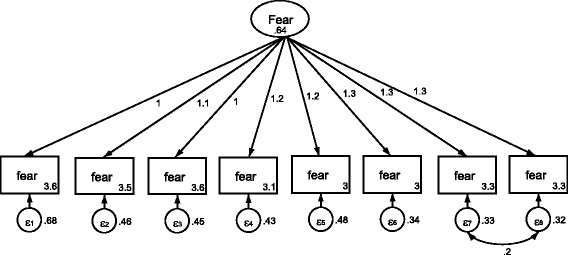
Standardized solution for the revised model based on confirmatory factor analysis of fear of breast cancer scale. Numbers in circles in rectangles indicate measurement errors

The confirmatory factor analysis model for the cancer fear scale can be seen in Table 5 and was as follows: *×* ^2^ = 420, CFI =0.80, TLI =0.72, RMSEA = 0.28 and SRMSR =0.06. The fit indices suggested a lack of fit with the data. In order to improve model fit, a correlated error term was added between items 7 and 8, which both related to intrusive thoughts. The final model provided a good fit to the data: *×* ^2^ *=* 86.33, CFI =0.99, TLI =0.99, RMSEA =0.04 and SRMSR =0.01 (Table 5; Fig. 2).

**Table 5:** The fit indexes of the initial and revised model of the confirmatory factor analyses for the Farsi version of the breast cancer fear scale (*N*=252)

Indexes Values							
	*x* ^2^	df	*x* ^2^/df	CFI	TLI	RMSEA	SRMSR
Initial model	420	20	21	0.80	0.72	0.28	0.06
Revised model	86.33	15	5.7	0.99	0.99	0.04	0.01

*Abbreviations*: *CFI* Comparative Fit Index, *RMSEA* Root Mean Square Error of Approximation, *SRMSR* Standardized Root Mean Square Residual, *TLI* Tucker Lewis Index

## Discussion

This study provided a cross-cultural adaptation and validation of mammography self-efficacy and breast cancer fear scales in Farsi for Iranian women. Processes of translation, back translation, and cultural adaptation of the questionnaires were conducted by experts. The results of this study provided initial evidence for the reliability and validity of both scales. This was a significant work resulting in considerable revisions to the original instruments, in particular to the mammography self-efficacy scale. This may be in part due to the influences of both culture and religion that result in non-applicability of these across different populations seamlessly [20]. While the psychometric properties of the mammography self-efficacy scale were previously evaluated by Hashemian in Sabzevar [20], the current work was conducted in Sanandaj that is a city in the western Iran with a distinct culture and religion from Sabzevar. As expected, the culture and religion in Sanandaj led to different results regarding mammography self-efficacy in comparison with previous study [20]. Our results were different on the 6 items relating to mammography self-efficacy. For example, the item “I can ask doctor to prescribe a mammography”. Bandura [11] identified verbal persuasion and social influence that would wary by cultures and can influence one’s self-efficacy as one of four sources that build self-efficacy [28]. A persuasive caregiver may be able to influence a woman’s decisions. Women in current sample might be more inclined to mammography by doctor’s recommendation. In addition, the items “I can take clothes off and not ashamed of it when [having a] mammogram” and “I can get mammograms despite of high aging [getting older]” that are influenced by culture and religion effects as other researchers have shown [29–31]. Investigating the other item “Even if I have a family history of breast cancer, I can get mammography [a mammogram]” and “although mammography is a little painful, I can do it” showed that having a family history of breast cancer, fear of finding a lump, or mammograms being painful cannot prevent women from getting a mammogram. This item related to physiological and affective states is a fourth source influences one’s self-efficacy which is in line with Bandura [28]. Bandura describes self-efficacy as having several dimensions. For example, self-efficacy beliefs may vary in strength. People may have strong or weak beliefs about their ability to perform specific steps related to a recommend behavior [28]. The item “Even if nobody comes to me, I can go to the mammography center” reflects a dimension of self-efficacy. The participants of current study perceived strength as the degree of certainty they have regarding their ability to get a mammogram. Finally, our results did not meet the factor loading criterion for the two items “talk to people at the mammogram center about my concern” and “sure can get a mammogram if I really want to” in Hashemian’s study.

MSS items loaded on factor 1 measured physiological states like having feelings of worry, pain, shame, being too old, and fear resulting from a breast cancer family history. Those items loaded on factor 2 measured one’s self-efficacy in the process of getting a mammogram. These results are in contrast with the finding Champion [18], Secginli [19], and Hashemian [20] in which all items loaded on a single factor. This contradictory result might be related to the additional items that our expert panel added to the instrument. Another explanation may be linked to cultural differences across study populations. The barriers that were added in the current study might not be considered as relevant for women that live in Sanandaj, but are for women in Turkey [19], the USA [12], and Sabzevar [20]. The panel of experts considered that all self-efficacy aspects should be dealt with in this screening behavior to the extent possible when considering the items. Bandura [10] suggested that measures of perceived self-efficacy should be tailored to the particular domain of functioning which is the object of interest. Furthermore, self-efficacy assessments must reflect the level of difficulty individuals believe they can overcome.

The adapted Farsi version of the self-efficacy for mammography scale consisted of 14 items. Most of the standardized factor loadings were moderate to large, suggesting that the majority of the observed variables were good measures of their latent construct. The standardized factor loadings ranged from 0.45 to 0.83 for the MSS items. These results are comparable with those reported in Champion’s study where factor loadings were within 0.37–0.76 [12]. The results of Secginli’s study showed a range from 0.62 to 0.82 and of Hashemian’s from 0.54 to 0.94 for the corresponding self-efficacy items [19, 20].

The Farsi version of the MSS had an internal consistency reliability comparable to previous versions. The Cronbach’s alpha coefficient for the MSS was 0.87, which is acceptable. In Champion’s and Secginli’s studies, in which the MSS included 10 items, the Cronbach’s alpha values were 0.94 [12, 18], and 0.90, respectively. Hashemian et al., reported Cronbach’s alpha of 0.90 for the MSS [20].

The Farsi version of the breast cancer fear scale in this study brought about results similar to the ones reported in the original English version by Champion in which all items loaded onto one factor [13], but they were not in agreement with the findings of Secginli in Turkey [19], where the items loaded onto 2 factors. In the current study, the standardized factor loadings ranged from 0.64 to 0.80. The breast cancer fear item loadings in our study were somewhat lower than those found in Champion’s study where factor loadings were within 0.75–0.86 [13], and in Secginli’s study were within 0.71 to 0.85 for the corresponding breast cancer fear items [19]. The Cronbach’s alpha coefficient for BCFS was 0.95. This result is comparable with those obtained by Champion and Secginli who reported Cronbach’s alpha coefficients as 0.94 and 0.90 [13, 19], respectively.

## Conclusion

This study provided a cross-cultural adaptation and validation of mammography self-efficacy and breast cancer fear scales in Farsi context. It is the first study where investigators tested the psychometric properties of a translated Farsi version of breast cancer fear among Iranian women. Mammography behavior should done in long intervals (once a year for women 50 or older), in contrast with lifestyle behaviors like exercise that should be done daily. Therefore, comprehensive psychometric work will help providing measures of perceived mammography self-efficacy that capture the distinct components of this concept. Future research are recommended to examine if MSS version can measure three dimensions of self-efficacy: generality, magnitude, and strength related to mammography behavior. Future research can test these instruments among women participating in mammography screening in a variety of health service settings or among women with different cultural backgrounds to increase the generalizability of the results.

## Abbreviations

BCFS: Breast Cancer Fear Scale
CBCFS: Champion’s Breast Cancer Fear Scale
CFA: Confirmatory factor analysis
CFI: Comparative Fit Index
CMSS: Champion’s Mammography Self-efficacy Scale
CVI: Content Validity Index
KMO: Kaiser-Meyer-Olkin
NDI: National Death Index
RMSEA: Root Mean Square Error of Approximation
SRMSR: Standardized Root Mean Square Residual
TLI: Tucker Lewis Index
